# Domiciliary pulse-oximetry at exacerbation of chronic obstructive pulmonary disease: prospective pilot study

**DOI:** 10.1186/1471-2466-10-52

**Published:** 2010-10-20

**Authors:** John R Hurst, Gavin C Donaldson, Jennifer K Quint, James JP Goldring, Anant RC Patel, Jadwiga A Wedzicha

**Affiliations:** 1 Academic Unit of Respiratory Medicine, Royal Free Campus, UCL Medical School, London, NW3 2PF, UK

## Abstract

**Background:**

The ability to objectively differentiate exacerbations of chronic obstructive pulmonary disease (COPD) from day-to-day symptom variations would be an important development in clinical practice and research. We assessed the ability of domiciliary pulse oximetry to achieve this.

**Methods:**

40 patients with moderate-severe COPD collected daily data on changes in symptoms, heart-rate (HR), oxygen saturation (SpO_2_) and peak-expiratory flow (PEF) over a total of 2705 days. 31 patients had data suitable for baseline analysis, and 13 patients experienced an exacerbation. Data were expressed as multiples of the standard deviation (SD) observed from each patient when stable.

**Results:**

In stable COPD, the SD for HR, SpO_2 _and PEF were approximately 5 min^-1^, 1% and 10l min^-1^. There were detectable changes in all three variables just prior to exacerbation onset, greatest 2-3 days following symptom onset. A composite Oximetry Score (mean magnitude of SpO_2 _fall and HR rise) distinguished exacerbation onset from symptom variation (area under receiver-operating characteristic curve, AUC = 0.832, 95%CI 0.735-0.929, p = 0.003). In the presence of symptoms, a change in Score of ≥1 (average of ≥1SD change in both HR and SpO_2_) was 71% sensitive and 74% specific for exacerbation onset.

**Conclusion:**

We have defined normal variation of pulse oximetry variables in a small sample of patients with COPD. A composite HR and SpO_2 _score distinguished exacerbation onset from symptom variation, potentially facilitating prompt therapy and providing validation of such events in clinical trials.

## Background

Exacerbations of chronic obstructive pulmonary disease (COPD) are deteriorations in respiratory health that punctuate the course of this prevalent condition [1]. Patients with COPD also experience day-to-day symptom variation. Distinguishing this from exacerbation is challenging but important as access to prompt treatment at exacerbation is associated with earlier recovery [2], while treating symptom variation as exacerbation might result in unnecessary exposure to therapy. There is real clinical need for an objective way to confirm exacerbation onset, which would also be important for validating these events in clinical trials. Current attention has focused on symptom scores to confirm exacerbation [3], but symptoms (even when scored) are, by definition, subjective. Despite exacerbations being associated with increases in local and systemic inflammation [4], no biomarker has been shown to reliably differentiate stable COPD from exacerbation [5].

Exacerbations are known to be associated with changes in physiological measures, such as peak-expiratory flow (PEF) [6], but absolute changes are small and could not reliably detect exacerbations when used in isolation [7]. We hypothesised that changes in heart rate and oxygen saturation (SpO_2_), readily measurable using pulse oximetry, might assist in the differentiation of stable COPD from exacerbation. Despite the wide availability of such devices, and their use in tele-monitoring programmes [8], there are no existing data examining day-to-day variation of oximetry variables in stable COPD, or the changes in heart rate and SpO_2 _observed through the onset and recovery of exacerbations.

We have performed a pilot study of daily domiciliary pulse oximetry in a patient sample from the London COPD cohort. Using prospective diary card data allowed us to precisely define the onset of exacerbation symptoms. We have therefore been able to describe the extent of normal variation in heart rate and SpO_2 _on symptom free days, the disease and demographic characteristics that affect this baseline variation, and the time-course of changes with exacerbation. We go on to report the ability of such data to differentiate exacerbation from both stable disease and day-to-day symptom variation.

## Method

### Subjects and Exacerbations

Subjects were recruited from the London COPD cohort, first established in 1995 to investigate the causes and mechanisms of exacerbation. The methodology has been previously described [6]. In brief, patients attend for regular clinical assessments to comprehensively phenotype their disease, and they complete daily diary cards. The diary cards include assessment of PEF (best of three attempts) and change in respiratory symptoms. Symptoms are only recorded when they are new or worse than usual. The symptoms are classified as major (breathlessness, sputum volume and sputum purulence) and minor (cough, wheeze, sore-throat and coryza). In this way we can monitor PEF and symptoms prior to, at the onset of, and during exacerbations.

Exacerbations were defined using criteria that we have previously validated against health-status [9], airway inflammatory markers [10], lung function decline [11] and physical activity [12]. As in these previous studies, exacerbations were defined as two or more days of ≥2 new or worsening symptoms, at least one of which must have been a major symptom. The first of these two days was the day of onset. An exacerbation was considered to have recovered on the first of two consecutive symptom free days. This methodology also allowed us to calculate each patient's exacerbation frequency over the 12-month period prior to recording pulse oximetry. The number of major and minor symptoms recorded on each exacerbation day was summed to give a 'Symptom Count', an assessment of exacerbation severity which can vary between zero and seven. As patients only record symptoms when they are new or worse than normal, the Symptom Count is usually zero in stable COPD. Days on which symptoms were recorded, but which did not reach our criteria for exacerbation (and were not during an exacerbation) were termed 'symptom days', representing day-to-day symptom variation. Days on which no additional symptoms had been recorded, the majority, were termed 'symptom free days'.

Patients were originally recruited from primary and secondary care and inclusion criteria included a post-bronchodilator Forced Expiratory Volume in 1 second (FEV_1_) <80% predicted, FEV_1_/FVC (Forced Vital Capacity) ratio <0.7, minimal or no reversibility to β_2_-agonists (<200 ml and/or 15%) and the absence of other significant respiratory disease. At recruitment, a full clinical assessment was performed, together with spirometry (volumetric storage spirometer 122, Vitalograph 2160, Maids Moreton, Buckingham, UK). The St. George's Respiratory Questionnaire (SGRQ) was used to assess health-status [13].

The study was approved by Royal Free Hampstead NHS Trust ethics committee, and all patients provided written informed consent.

### Domiciliary Pulse Oximetry

Twenty-eight Nonin 9500 Onyx™ pulse oximeters (Plymouth, Minnesota, US) were rotated through 40 patients between September 2008 and February 2010 (a convenience sample of those attending for routine clinic appointments). The equipment complies with ISO 10993-1 (Biological Evaluation of Medical Devices) and has a manufacturer-stated accuracy of +/-3 min^-1 ^within the range 20-250 min^-1 ^and +/-2% SpO_2 _(compared to invasive assessment) within the range 70-100%. All the patients were in sinus rhythm, none had respiratory failure, and none were using supplementary oxygen or ventilatory support.

Patients were asked to record their daily heart rate and SpO_2 _on a modified version of our standard diary card (in addition to changes in symptoms and PEF). Patients were given written instructions reminding them to take the oximetry reading at the same time each morning, before morning medication, before performing the PEF test, from the same finger, and after sitting for ten minutes at rest. Patients were asked to record the highest value obtained during each measurement.

### Statistical Analysis

Data were analysed using SPSS version 14.0 http://www.spss.com. Normally distributed data are reported as mean and standard deviation (SD), non-parametric data as median and interquartile range (IQR). Comparisons between groups employed t-test or ANOVA as described in the Results. Correlations employed Pearson and Spearman techniques.

We first assessed the extent of day-to-day variation in heart rate, SpO_2 _and PEF. To do this, we calculated the individual mean and SD of these variables in the stable (symptom free) state. After initial distribution of the oximeters to each patient, the first seven days of data were ignored, to allow each patient to develop an oximetry measurement routine. The mean and SD of the heart rate, SpO_2 _and PEF were then calculated for the next 30 symptom free days (these data were normally distributed). For this analysis, data from seven days preceding any exacerbation to 14 days following exacerbation recovery were excluded. Symptom days were also ignored. On 34 otherwise stable days (3.7%), in 30 of the 31 patients, data were incomplete necessitating the inclusion of additional days such that each patient had thirty stable days monitoring of all three variables (the final patient had a prolonged period in which oxygen saturation was not recorded, prior to re-education). A period of 30 days was chosen after initial inspection of the data revealed stabilisation of the SD took up to three weeks. This methodology implies that on subsequent baseline (symptom free) days, 95% of values would be expected to lie between the mean +/- 1.96 (approximately 2) SD.

We next examined the time course of changes in heart rate, SpO_2 _and PEF in prospectively observed exacerbations, in those patients with stable baseline data. Data were examined from seven days prior, to seven days following the onset of exacerbation symptoms. There were 13 exacerbations in 13 subjects for this analysis, which is therefore based on 195 patient-days of exacerbation monitoring.

The data in individual patients were first expressed as multiples of that patient's SD. This is calculated using the formula (V_D_-V_M_)/V_SD_, where the measurement on that day, stable mean and stable standard deviation of each variable V (heart rate, oxygen saturation and PEF) are indicated by V_D_, V_M _and V_SD _respectively. For example, if a patient's mean (SD) heart rate when stable was 75 (5) min^-1^, and the heart rate on the day of onset was 85 min^-1^, the value recorded would be (85-75)/5 = +2. If the patient's heart rate had been 70 min^-1^, the value recorded would be (70-75)/5 = -1.

Next, we wished to explore whether changes in oximetry variables with or without PEF could reliably distinguish exacerbation onset from other days, particularly day-to-day symptom variation (symptom days). Given that exacerbations were typically associated with falls (rather than rises) in SpO_2 _and PEF, and a rise (rather than fall) in heart rate, we hypothesised that a composite score might perform better than the individual measurements alone. In preliminary analysis, we found no evidence for a significant correlation between SpO_2 _and heart rate in the stable state and therefore considered these variables independent. There was also no significant correlation between the greatest change in heart rate and greatest change in oxygen saturation over the first two exacerbation days, suggesting these variables were indeed independent (r = -0.32, p = 0.28). We therefore calculated an Oximetry Score (positive magnitude in SD units of the fall in SpO_2 _and rise in heart rate, /2) and an Oximetry-PEF Score (positive magnitude in SD units of the fall in SpO_2_, fall in PEF and rise in heart rate, /3).

**Figure 1:**
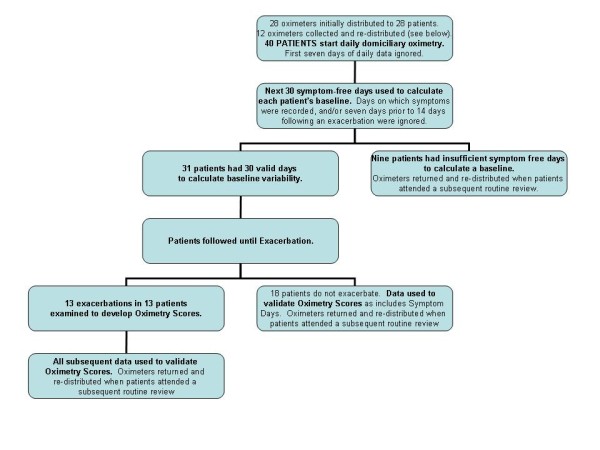
**Study Profile**. Study profile summarising distribution of oximeters and data collection. No data were used in more than one analysis.

We next assessed the ability of the Oximetry and Oximetry-PEF scores to differentiate exacerbation onset days from other days, in particular days on which symptom variation had been recorded (symptom days). To do this, we used all the remaining data that had not formed part of the above analyses (see Figure 1). Days were classified as symptom free, symptom, or exacerbation onset as described above. At exacerbation onset, the day with the higher score of the first two days was selected. All other exacerbation days were ignored. This analysis is based on 1580 days of follow-up (1469 symptom free days, 104 symptom days, and seven exacerbation onset days).

Receiver-operating characteristic (ROC) curves were constructed to assess the performance of oximetry indices and PEF in differentiating exacerbation onset from other days, and day-to-day symptom variation (symptom days). The test with the greatest area under the ROC curve was taken to have the best performance. We report sensitivity and specificity, but not positive predictive values as these would be dependent on the prevalence of the outcome (exacerbation) in this particular dataset.

## Results

### Defining Normal Variation of Pulse Oximetry variables and PEF in Stable COPD

The study profile is illustrated as Figure 1 which shows that of the 40 patients given oximeters, 31 had sufficient symptom and exacerbation free data for analysis in the stable state. Their characteristics are reported in Table 1. Their mean age was 72 years, 21 were male and their mean FEV_1 _was 1.10l (45.9% predicted). The baseline data in Table 1 were collected at routine (stable) clinic visits closest to the start of the oximetry recording.

**Table 1:** Clinical characteristics, and results of 30 days stable state monitoring in 31 patients with COPD.

	Mean	SD
Age (years)	72.1	6.9
FEV_1 _(l)*	1.10	0.49
FEV_1 _(% predicted)*	45.9	22.7
FVC (l)*	2.46	0.82
FEV_1_/FVC*	0.45	0.15
Body Mass Index (BMI) (kgm^-2^)	27.0	5.6
St. George's Respiratory Questionnaire (SGRQ) (Total Score)	49.9	16.0

	**Median**	**IQR**
Smoking (Pack years)	51	38-66
Exacerbation Frequency**	3	1-4

	**n**	**%**
Male	21	68
Current Smokers	6	19
β-blocker or rate-limiting Calcium-channel antagonist	7	23

	**Mean**	
Stable Mean Heart Rate (min^-1^)	77.8	
Stable SD Heart Rate (min^-1^)	4.9	
Stable Mean Oxygen Saturation (SpO_2_) (%)	94.8	
Stable SD Oxygen Saturation (SpO_2_) (%)	1.0	
Stable Mean Peak Expiratory Flow (PEF) (lmin^1^)	231.5	
Stable SD Peak Expiratory Flow (PEF) (lmin^1^)	11.4	

*: post-bronchodilator.

**: the number of symptom-defined exacerbations over the previous year of the study.

Of the remaining nine patients, 7/40 (17.5%) did not have 30 days clear of all symptoms, despite prolonged recording (median 175 days, approximately six months). One patient received the oximeter late in January 2010, and one patient did not record PEF correctly.

Table 1 also reports the mean of the individual patient mean and SD for the pulse oximetry variables and PEF. These data were normally distributed and the analysis is based on 930 patient-days of follow-up. The mean of the individual SD for heart rate, SpO_2 _and PEF, measures of day-to-day variation in the stable state, were approximately 5 min^-1^, 1% and 10l min^-1 ^respectively. Therefore, on 95% of stable (symptom free) days patients should have readings within approximately 10 min^-1^, 2% and 20l min^-1 ^above and below their stable mean value.

We next went onto explore whether day-to-day variation in heart rate, SpO_2_, and PEF was affected by patient or COPD characteristics. These data are reported in Table 2. The variation in heart rate, SpO_2 _and PEF was not affected by disease severity (FEV_1 _% predicted), smoking status or exacerbation frequency. As expected, older patients had reduced variation in heart rate (r = -0.39, p = 0.030). The variation in SpO_2 _was statistically, but not clinically greater in women (1.2 vs. 0.9%, p = 0.027). The mean and SD heart rate were not significantly different between patients who were and were not prescribed β-blocker or rate limiting calcium-channel antagonist drugs (73 vs. 79 min^-1 ^p = 0.215, and 4.8 vs. 4.9 min^-1 ^p = 0.883 respectively; t-test). All the patients had already been prescribed long-acting bronchodilators (β_2_-agonist and/or anti-cholinergics).

**Table 2:** Variation in, and absolute stable Heart Rate, Oxygen Saturation and peak-expiratory flow (PEF) by patient and COPD disease characteristics; n = 31.

	**Age**^**1**^	**Sex F vs. M**^**2**^	**Current Smoker vs. Not**^**2**^	**FEV**_**1 **_**(% predicted)**^**1**^	**Exacerbation Frequency (previous yr)**^**3**^
**Mean Heart Rate (min**^**-1**^**)**	r = -0.28	80 vs. 77	77 vs. 78	r = -0.26	rho = 0.23
	p = 0.134	p = 0.525	p = 0.862	p = 0.164	p = 0.207
**SD Heart Rate (min**^**-1**^**)**	r = -0.39	4.7 vs. 5.0	4.5 vs. 4.9	r = -0.35	rho = -0.05
	p = 0.030*	p = 0.724	p = 0.713	p = 0.054	p = 0.797
**Mean Oxygen Saturation (%)**	r = 0.08	94 vs. 95	95 vs. 95	r = 0.14	rho = 0.21
	p = 0.688	p = 0.358	p = 0.644	p = 0.470	p = 0.266
**SD Oxygen Saturation (%)**	r = 0.17	1.2 vs. 0.9	1.0 vs. 1.0	r = -0.21	rho = -0.03
	p = 0.350	p = 0.027*	p = 0.769	p = 0.267	p = 0.862
**Mean PEF (lmin**^**-1**^**)**	r = -0.03	198 vs. 248	258 vs. 225	r = 0.25	rho = 0.09
	p = 0.858	p = 0.085	p = 0.378	p = 0.172	p = 0.647
**SD PEF (lmin**^**-1**^**)**	r = -0.02	12 vs. 11	10 vs. 12	r = 0.10	rho = 0.00
	p = 0.921	p = 0.834	p = 0.446	p = 0.608	p = 0.982

(Statistics: ^1^= Pearson correlation. ^2^= t test. ^3^= Spearman correlation).

### Time Course of Changes in Heart Rate, Oxygen Saturation and PEF prior to, at onset, and with recovery of Exacerbation

**Figure 2:**
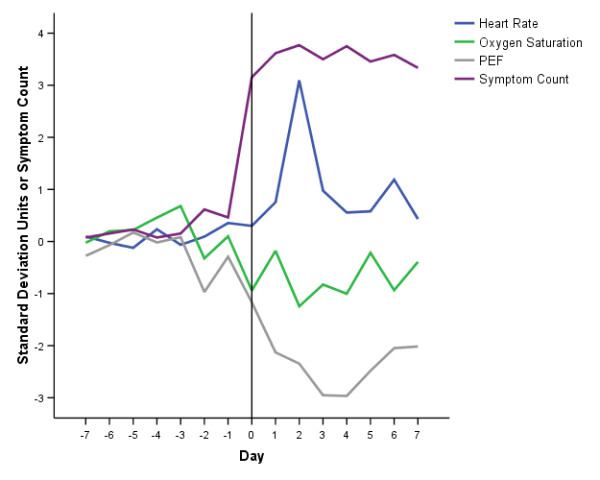
**Time Course of Symptoms and Oximetry Variables at Exacerbation**. Time course of symptom count, heart rate, oxygen saturation (SpO_2_) and peak-expiratory flow (PEF) from seven days prior (-7) to exacerbation onset (day 0, vertical line) and seven days into exacerbation of COPD. Values of the physiological variables are the mean of 13 exacerbations in 13 patients, expressed as standard deviation multiples away from each patient's stable mean (see text for details). A 1SD change is an approximate alteration in heart rate of 5 min^-1^, SpO_2 _of 1% and PEF of 10l min^-1^. Symptom count values are the mean from the 13 patients.

**Figure 3:**
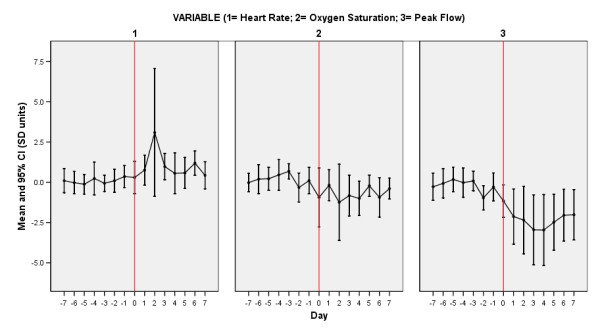
**Time Course of Symptoms and Oximetry Variables At Exacerbation**. Time course of heart rate, oxygen saturation (SpO_2_) and peak-expiratory flow (PEF) from seven days prior (-7) to exacerbation onset (day 0, vertical line) and seven days into exacerbation of COPD. Data expressed as mean and 95%CI (in SD units) for each variable separately.

Figures 2 and 3 report the time course of the oximetry variables and PEF, and the Symptom Count, through the prodrome, onset and recovery with treatment of 13 exacerbations in 13 patients (12 treated, one hospitalised, median (IQR) delay between symptom onset and treatment 5 (3-6) days).

The averaged data (which would not be expected to vary significantly from zero in the baseline state) suggest increased variation in heart rate, SpO_2 _and PEF immediately preceding exacerbation, a rise in heart rate, fall in SpO_2 _and fall in PEF at exacerbation onset, and further changes which are maximal at 2 days, 2 days and 3-4 days into the exacerbation respectively. The maximum magnitude of the change in SpO_2 _(-1.24SD, day 2) was smaller than the changes in heart rate (+3.09SD, day 2) and PEF (-2.97SD, day 4).

**Figure 4:**
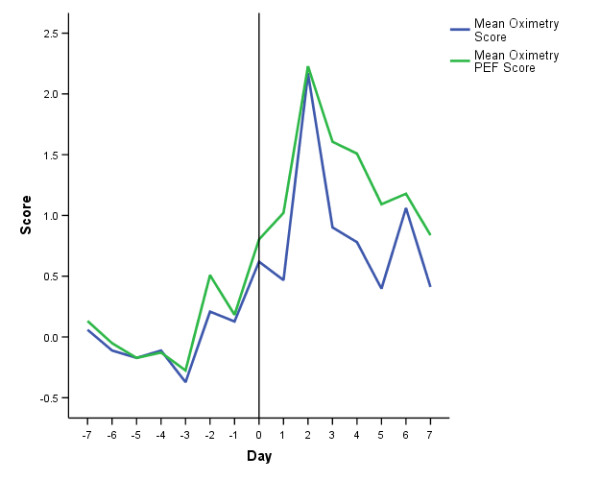
**Time Course of Oximetry and Oximetry-PEF Scores At Exacerbation**. Time course of Oximetry and Oximetry-PEF scores from seven days prior (-7) to exacerbation onset (day 0, vertical line) and seven days into exacerbation of COPD. Values are the mean score of 13 exacerbations in 13 patients (see text for details).

**Figure 5:**
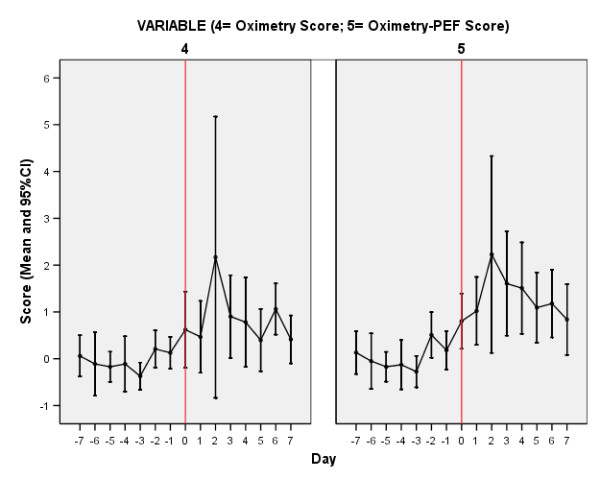
**Time Course of Oximetry and Oximetry-PEF Scores At Exacerbation**. Time course of Oximetry and Oximetry-PEF scores from seven days prior (-7) to exacerbation onset (day 0, vertical line) and seven days into exacerbation of COPD. Data represent the mean and 95%CI for each score separately.

Figures 4 and 5 report the time course of the composite scores for the same exacerbations over the same time period The scores appear to perform similarly, both showing a tendency to rise on the days prior to exacerbation onset, and peaking at exacerbation day two (the third day of exacerbation symptoms). The peak values (day two) were 2.17 (Oximetry Score) and 2.23 units (Oximetry-PEF Score).

### Ability of oximetry variables and PEF to differentiate exacerbation onset from stable (symptom free) days and symptom days

There were significant differences using ANOVA in the means of both the Oximetry and the Oximetry-PEF Scores between symptom free, symptom and exacerbation onset days: Oximetry Score (0.22 vs. 0.40 vs. 1.54 respectively, p = 0.002); Oximetry-PEF Score (0.17 vs. 0.92 vs. 2.14 respectively, p < 0.001).

We calculated the area under ROC curves to inform on the ability of the individual component variables and two scores to confirm exacerbation onset. Table 3 reports the results in differentiation of exacerbation onset days from any other day (symptom and symptom free). This assesses the ability to identify exacerbation onset in the absence of an assessment of symptoms, providing truly objective confirmation of exacerbation. The two composite scores performed better than the individual variables (higher AUC), significantly better than by chance, and both had AUC greater than the commonly accepted criteria for validity of 0.8.

**Table 3:** Ability of the individual components and composite scores to differentiate exacerbation onset days from all other days.

	AUC	95%CI	p =
**Heart Rate**	0.819	0.754-0.884	0.004
**Oxygen Saturation**	0.712	0.557-0.866	0.053
**Peak-Expiratory Flow (PEF)**	0.805	0.655-0.956	0.005
**Oximetry Score**	0.849	0.787-0.912	0.001
**Oximetry-PEF Score**	0.897	0.839-0.955	<0.001

p value represents difference from chance (AUC = 0.5).

**Figure 6:**
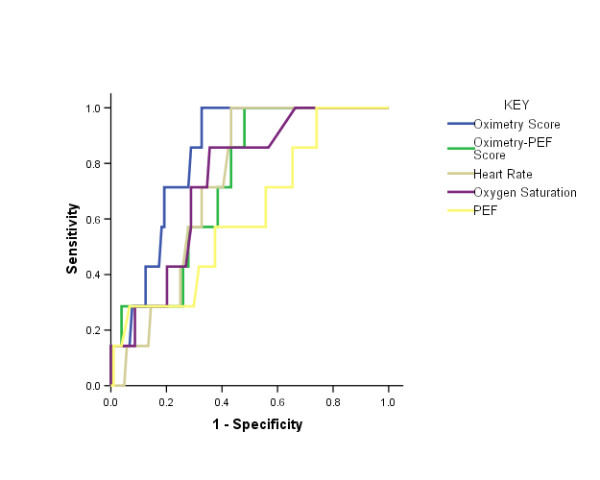
**ROC Curves**. ROC curves for the ability of the individual components, and Oximetry and Oximetry-PEF scores to differentiate exacerbation onset from day-to-day symptom variation (symptom days) in COPD. The data are reported in Table 4.

Table 4 reports the results of an analysis, illustrated in Figure 6, assessing the ability of the components and composite scores to differentiate exacerbation onset days from day-to-day symptom variation (symptom days). Notably, the mean and 95%CI values of the Oximetry-PEF score were all above zero from the day of exacerbation onset through to day seven. The Oximetry score performed better, but not significantly better than the Oximetry-PEF score (AUC 0.832 vs. 0.732, p = 0.20).

**Table 4:** Ability of the individual components and composite scores to differentiate exacerbation onset days from symptom days (day-to-day symptom variation).

	AUC	95%CI	p =
**Heart Rate**	0.731	0.613-0.849	0.042
**Oxygen Saturation**	0.740	0.590-0.889	0.034
**Peak-Expiratory Flow (PEF)**	0.615	0.410-0.819	0.311
**Oximetry Score**	0.832	0.735-0.929	0.003
**Oximetry-PEF Score**	0.732	0.589-0.876	0.040

p value represents difference from chance (AUC = 0.5).

The sensitivity and specificity of the Oximetry Score in differentiating exacerbation onset from day-to-day symptom variation is reported in Table 5. A + 1SD change in score could represent, for example, a fall in oxygen saturation of 1% together with a rise in heart rate of 5 min^-1^. In the presence of symptoms, such a change had a modest sensitivity of 71% and specificity of 74% for the presence of exacerbation. On a stable day, the chance of a 1SD fall in SpO_2 _and a 1SD rise in heart rate both occurring would be approximately 2.5% (by definition, 68.2% of stable days lie within mean +/- 1SD for each variable, therefore on (100-68.2)/2 = 15.9% of days this is greater than 1SD in the specified direction, and therefore the chance of both occurring together = 0.159 × 0.159 = 2.5% days).

**Table 5:** Sensitivity and specificity of the Oximetry Score in differentiating exacerbation onset from symptom days (day-to-day symptom variation) in COPD.

Oximetry Score*	Sensitivity	Specificity
**+0.5**	100%	54%
**+1.0**	71%	74%
**+1.5**	29%	89%
**+2.0**	14%	94%

*A +1SD change in score could represent, for example, a fall in oxygen saturation of 1% and a rise in heart rate of 5 min^-1^, or no change in oxygen saturation from baseline and a rise in heart rate of 10 min^-1^. See text for further details.

## Discussion

We have provided a preliminary demonstration that changes in heart rate and oxygen saturation, measured using domiciliary pulse oximetry and incorporated into a composite score, are able to differentiate exacerbation onset from stable COPD with reasonable sensitivity and specificity. In addition, we have defined the extent of daily variation in oximetry variables in stable, ambulatory patients with COPD, and demonstrated changes in these indices immediately prior to, at onset and through the recovery of an exacerbation. Our results and methodology will be of immediate interest to those employing domiciliary oximetry to monitor COPD and, with further validation, have the potential to facilitate objective confirmation of exacerbation using simple technology, enabling access to prompt therapy and verification of events in clinical trials.

Our study has a number of strengths. By prospectively recording daily symptom changes we have been able to differentiate symptom free days from day-to-day symptom variation, and by using validated criteria for exacerbation we have been able to differentiate symptom variation from exacerbation onset. In total, the data reported in this manuscript comprises 2705 days (7.4 patient-years) recording symptoms, oximetry and PEF in 31 patients with well-characterised COPD. These patients are typical of those in whom monitoring and early therapy at exacerbation may be desirable, having moderate and severe disease and a previous history of exacerbation.

Exacerbations are important events that punctuate the natural history of COPD. Patients experiencing frequent exacerbations have a more rapid decline in lung function [11,14], poorer quality-of-life [9], reduced physical activity [12] and greater mortality [15]. Exacerbations are therefore associated with a considerable burden of health-care costs [16] such that preventing exacerbations is a key goal of COPD therapy. Prompt therapy is associated with more rapid exacerbation recovery [2] and there is therefore the need to identify exacerbations early in their course. This has resulted in attempts to objectively confirm exacerbations, using tools such as the EXACT-PRO [3], but quantifying symptoms does not address the subjectivity of symptoms themselves. Symptom scores may also be prone to ceiling effects, and exacerbation symptoms must be differentiated from the day-to-day symptom variations that characterise COPD. Exacerbations remain difficult events to detect as there is currently no diagnostic biomarker [5]. This can result in over-treating symptom variation as exacerbation, and delaying exacerbation therapy when deteriorations are mis-attributed to symptom variation. This may be particularly important during high-risk periods for recurrent exacerbations, in the weeks immediately following a first event [17]. The distinction between stable disease and exacerbation is not just important for patients, but is also relevant in the conduct of clinical trials where the ability to objectively confirm these events would be a major development.

We have demonstrated changes in heart rate, SpO_2 _and PEF immediately prior to, at onset and during exacerbations of COPD. A composite score of change in heart rate and SpO_2 _performed moderately well in distinguishing day-to-day symptom variation from COPD exacerbation. This is a potentially important development. Oximetry is cheap and reliable, and electronic data capture make calculation of composite scores practical. With knowledge of a patient's baseline heart rate and SpO_2_, calculation of the score can distinguish symptom variation from exacerbation onset by the second day of symptom changes, with a cut-off that can be chosen to maximise sensitivity or specificity. In this way, inappropriate antibiotics might be avoided, and early therapy might be facilitated. Key to our approach was the careful collection of data from each patient at baseline, and expression of subsequent changes as multiples of that patient's own, individual day-to-day variation.

Although pulse oximetry is widely used in tele-monitoring programs [8], and by patients themselves, we are not aware of any previous work defining the extent of day-to-day variation in heart rate and oxygen saturation in patients with moderate and severe COPD. Indeed, the European Commission Communication on Telemedicine has highlighted the need for robust methodological studies in this area [18], such as ours. Use of oximeters supports the current emphasis on management of long-term conditions through self-management and care closer to (or in) the patient's home. Even in the absence of telemedicine, our data could be incorporated into a written self-management plan. For example, we have shown that there would be only a 2.5% chance of a patient having an oxygen saturation <2% of their baseline mean on a stable day, and a reduction in saturation of this magnitude, or rise in heart rate >10 min^-1^, might be used to prompt re-assessment of respiratory health. Our technique, employing simple, readily available technology is much more practical than the domiciliary measurement of airway or systemic biomarkers which in any case have been unable to differentiate exacerbation from the stable state [5].

There are some limitations of our approach. We present our findings as preliminary but believe these data will be of immediate utility to those monitoring patients with domiciliary oximetry, enabling subsequent validation of our technique in larger cohorts. To establish an individual patient's baseline, our method requires rigorous data collection, at the sensitivity limit of currently available technology. However, with tele-health such methods are increasingly practical. We did not include patients with respiratory failure and it may be that oxygen saturation changes at exacerbation in patients on the steeper part of the oxygen dissociation curve are greater. Patients in atrial fibrillation were also excluded. We do not have data to inform on how long day-to-day variation in the baseline state remains stable. We have previously reported that PEF declines (slowly) with time [11] and we are not able to state how frequently the stable monitoring period would need to be repeated. Our data do not inform on the ability of pulse oximetry to distinguish exacerbation of COPD from other causes of symptom deteriorations in these patients (such as pneumonia), and it is quite likely that such pathologies would also result in heart rate and SpO_2 _changes. Exacerbation of COPD remains a clinical diagnosis of exclusion and the clinician (and patient) must remain aware that other conditions may mimic or complicate exacerbations [19]. We note that there were fewer unreported exacerbations in this study than we have previously detected from this cohort. Finally, it is important to remark that in some patients (17.5% in this study) it was not possible to ascertain baseline readings on 30 symptom free days, despite prolonged monitoring, and this might limit applicability to a proportion of patients in clinical practice.

The Oximetry Score was able to differentiate exacerbation onset from day-to-day symptom variation with reasonable sensitivity and specificity. Our data are the first to report that day-to-day symptom variation in COPD is associated with small, but demonstrable changes in physiology. Addition of PEF to the oximetry variables was not associated with a better composite score, despite demonstrating detectable changes in PEF at exacerbation (as previously reported [6]). This may be because it is more difficult to assess smaller changes in PEF, on a scale where 10l min^-1 ^is the minimal detectable difference. In addition, the PEF changes lag those in heart rate and SpO_2 _and were maximal at four days into the exacerbation (symptom day five). One attraction of adding PEF is that this is a marker of airway involvement, believed to be the primary site of pathology during exacerbations. In contrast SpO_2 _reflects ventilation-perfusion relationships, and changes in heart rate a variety of mechanisms including increased β_2_-agonist use. We do not have data on β_2_-agonist use, though this would not be expected to significantly affect changes in oxygen saturation or PEF. Adding PEF increased the performance of the score differentiating exacerbation onset from all other days, regardless of symptoms, but PEF is also effort dependent and given the difficulties in assessing PEF reliably, and the small changes involved, an approach restricted to heart rate and SpO_2 _would seem to be more attractive. This is in contrast to the validity of PEF during exacerbations of asthma [20].

Both scores, and individual components showed increased variation immediately preceding exacerbation onset, with changes that were greatest in magnitude 2-4 days following the start of exacerbation symptoms (at about the time that patients commenced therapy). The magnitude of the changes in heart rate and PEF were greater than the magnitude of change in SpO_2_, such that many exacerbations are associated with only small (or no) change(s) in SpO_2_. Much of the initial signal in the composite scores may therefore be attributed to alterations in heart rate. As initial changes were small, it seems likely that exacerbation detection should remain dependent on patients first identifying changes in symptoms and our data suggest that oximetry can assist in differentiating exacerbation symptoms from day-to-day variation. However, we have also provided evidence that the composite scores can identify exacerbation onset from all other days in the absence of symptom assessment. The Oximetry-PEF score performed best in this instance. Oximetry monitoring may therefore also be important for encouraging assessment of 'unreported' (and therefore untreated) exacerbations [6,21], events which contribute to the negative impact of exacerbations on COPD health status.

## Conclusion

In summary, this is the first report to provide data on the day-to-day variation of heart rate and oxygen saturation in ambulatory patients with stable moderate and severe COPD. We have further described how this information might be used to facilitate confirmation of COPD exacerbation onset, desirable in facilitating access to prompt treatment, and providing objective confirmation of these events in the context of clinical trials. These data will be of immediate use to those employing domiciliary pulse oximetry monitoring in COPD, and there is now the need to replicate these findings in separate, larger cohorts with the aim of using oximetry to facilitate and guide exacerbation therapy.

## Abbreviations

AUC: Area under Curve; COPD: Chronic Obstructive Pulmonary Disease; FEV_1_: Forced Expiratory Volume in 1 second; FVC: Forced Vital Capacity; HR: Heart Rate; PEF: Peak Expiratory Flow; ROC: Receiver-Operating Characteristic; SD: Standard Deviation; SpO_2_: Oxygen Saturation.

## Competing interests

The authors declare that they have no competing interests.

## Authors' contributions

JRH conceived the study. All authors contributed to the study design. Data were collected and collated by JQ, JJP, ARC and GCD. JRH conducted the primary analysis. All authors were involved in interpretation of the data. JRH wrote the first draft of the manuscript. All authors critically reviewed the manuscript and have seen and approved the final version.

## Pre-publication history

The pre-publication history for this paper can be accessed here:

http://www.biomedcentral.com/1471-2466/10/52/prepub
